# Supplementary material for: Usability of augmented reality technology in tele-mentorship for managing clinical scenarios—A study protocol
Source: PLoS One. 2022 Mar 31;17(3):e0266255. doi: 10.1371/journal.pone.0266255 (PMC8970358; doi:10.1371/journal.pone.0266255)
Supplement: S1 File — (PDF) [file pone.0266255.s001.pdf]

Research Integrity and Ethics Unit

UNIVERSITY of TASMANIA

Ethics Approval Letter

06/10/2021

To: AssocProf Barnett

Project ID: 23343

Project Title: Usability of Augmented Reality technology in situational tele-mentorship for managing clinical scenarios: A feasibility study

The amendment received in support of the above named project has been approved by the University of Tasmania Human Research Ethics Committee on 06.10.2021.

Approval has been granted for the following documentation:

Submission Document Name	Submission Document File Name	Submission Document Type	Submission Document Date	Submission Document Version
Responses to reviewers comments 10.12.20	Responses to reviewers comments 10.12.20.docx	EVIDENCE OF THE OUTCOME	10/12/2020	1
School approval 11.12.20	School approval 11.12.20.png	EVIDENCE OF THE OUTCOME	11/12/2020	1
Protocol_Project Description - Clean - 20210927	Protocol_Project Description - Clean - 20210927.docx	PROTOCOL	27/09/2021	4
Appendix 6 - AR usability assessment - Mentors - Tracked - 20210707	Appendix 6 - AR usability assessment - Mentors - Tracked - 20210707.docx	QUESTIONNAIRE	07/07/2021	2
Appendix 7 - AR usability assessment - Mentees - Tracked - 20210707	Appendix 7 - AR usability assessment - Mentees - Tracked - 20210707.docx	QUESTIONNAIRE	07/07/2021	2
Appendix 8 - Mentorship Effectiveness Scale - Mentors - Tracked - 20210707tb	Appendix 8 - Mentorship Effectiveness Scale - Mentors - Tracked - 20210707tb.docx	QUESTIONNAIRE	07/07/2021	2
Appendix 20 - General information - 20210927	Appendix 20 - General information - 20210927.docx	QUESTIONNAIRE	27/09/2021	1
Appendix 22 - Evaluation of self-confidence - HE scenario - 20210927	Appendix 22 - Evaluation of self-confidence - HE scenario - 20210927.docx	QUESTIONNAIRE	27/09/2021	1
Appendix 23 - Skill performance checklist - HE scenario - 20210927	Appendix 23 - Skill performance checklist - HE scenario - 20210927.docx	QUESTIONNAIRE	27/09/2021	1
Appendix 21 - Scenario HE - 20210927	Appendix 21 - Scenario HE - 20210927.docx	OTHER PROJECT-RELATED DOCUMENTATION	27/09/2021	1
Appendix 18 - Advert for mentee recruitment - Tracked - 20210927	Appendix 18 - Advert for mentee recruitment - Tracked - 20210927.pptx	ADVERTISING MATERIAL	27/09/2021	3
Appendix 18 - Advert for mentee recruitment - Clean - 20210927	Appendix 18 - Advert for mentee recruitment - Clean - 20210927.pptx	ADVERTISING MATERIAL	27/09/2021	3
Appendix 6 - AR usability assessment - Mentors - Clean - 20210707	Appendix 6 - AR usability assessment - Mentors - Clean - 20210707.docx	QUESTIONNAIRE	07/07/2021	2
Appendix 7 - AR usability assessment - Mentees - Clean - 20210707	Appendix 7 - AR usability assessment - Mentees - Clean - 20210707.docx	QUESTIONNAIRE	07/07/2021	2
Appendix 8 - Mentorship Effectiveness Scale - Mentors - Clean - 20210707tb	Appendix 8 - Mentorship Effectiveness Scale - Mentors - Clean - 20210707tb.docx	QUESTIONNAIRE	07/07/2021	2

Protocol_Project Description - Tracked - 20210927	Protocol_Project Description - Tracked - 20210927.docx	PROTOCOL (TRACKED)	27/09/2021	4
Appendix 1 - Participant Information Statement & Consent Form - Mentors - Tracked - 20211005	Appendix 1 - Participant Information Statement & Consent Form - Mentors - Tracked - 20211005.docx	PARTICIPANT INFORMATION AND CONSENT FORM	05/10/2021	4
Appendix 1 - Participant Information Statement & Consent Form - Mentors - Clean - 20211005	Appendix 1 - Participant Information Statement & Consent Form - Mentors - Clean - 20211005.docx	PARTICIPANT INFORMATION AND CONSENT FORM	05/10/2021	4
Appendix 2 - Participant Information Statement & Consent Form - Mentees - Clean - 20211005	Appendix 2 - Participant Information Statement & Consent Form - Mentees - Clean - 20211005.docx	PARTICIPANT INFORMATION AND CONSENT FORM	05/10/2021	5
Appendix 2 - Participant Information Statement & Consent Form - Mentees - Tracked - 20211006	Appendix 2 - Participant Information Statement & Consent Form - Mentees - Tracked - 20211006.docx	PARTICIPANT INFORMATION AND CONSENT FORM	06/10/2021	5
Response to ERM Ethics Committee Reviewer Comments 20211006	Response to ERM Ethics Committee Reviewer Comments 20211006.docx	OTHER PROJECT-RELATED DOCUMENTATION	06/10/2021	1

The University of Tasmania Human Research Ethics Committee has provided approval for the project to be conducted at the following sites:

Newnham campus Simulation and Clinical education training centre, School of Nursing

Please ensure that all investigators involved with this project have cited the approved versions of the documents listed within this letter and use only these versions in conducting this research project.

This approval constitutes ethical clearance by the University of Tasmania Human Research Ethics Committee. The decision and authority to commence the associated research may be dependent on factors beyond the remit of the ethics review process. For example, your research may need ethics clearance from other organisations or review by your research governance coordinator or Head of Department. It is your responsibility to find out if the approvals of other bodies or authorities are required. It is recommended that the proposed research should not commence until you have satisfied these requirements.

In accordance with the National Statement on Ethical Conduct in Human Research, it is the responsibility of institutions and researchers to be aware of both general and specific legal requirements, wherever relevant. If researchers are uncertain they should seek legal advice to confirm that their proposed research is in compliant with the relevant laws. University of Tasmania researchers may seek legal advice from Legal Services at the University.

The University of Tasmania Human Research Ethics Committee (HREC) operates under and is required to comply with the National Statement on the Ethical Conduct in Human Research.

Therefore, the Chief Investigator's responsibility is to ensure that:

(1) All investigators are aware of the terms of approval, and that the research is conducted in compliance with the HREC approved protocol or project description.

(2) Modifications to the protocol do not proceed until **approval** is obtained in writing from the HREC. This includes, but is not limited to, amendments that:

(i) are proposed or undertaken in order to eliminate immediate risks to participants;
(ii) may increase the risks to participants;
(iii) significantly affect the conduct of the research; or
(iv) involve changes to investigator involvement with the project.

Please note that all requests for changes to approved documents must include a version number and date when submitted for review by the HREC.

(3) Reports are provided to the HREC on the progress of the research and any safety reports or monitoring requirements as indicated in NHMRC guidance.

Guidance for the appropriate forms for reporting such events in relation to clinical and non-clinical trials and innovations can be located under the ERM "Help Tab" in "Templates". All adverse events must be reported regardless of whether or not the event, in your opinion, is a direct effect of the therapeutic goods being tested.

(4) The HREC is informed as soon as possible of any new safety information, from other published or unpublished research, that may have an impact on the continued ethical acceptability of the research or that may indicate the need for modification of the project.

(5) All research participants must be provided with the current Participant Information Sheet and Consent Form, unless otherwise approved by the Committee.

(6) This study has approval for four years contingent upon annual review. A Progress Report is to be provided on the anniversary date of your approval. Your first report is due on the anniversary of your approval**,** and you will be sent a courtesy reminder closer to this due date. Ethical approval for this project will lapse if a Progress Report is not submitted in the time frame provided.

(7) A Final Report and a copy of the published material, either in full or abstract, must be provided at the end of the project.

(8) The HREC is advised of any complaints received or ethical issues that arise during the course of the project.
(9) The HREC is advised promptly of the emergence of circumstances where a court, law enforcement agency or regulator seeks to compel the release of findings or results. Researchers must develop a strategy for addressing this and seek advice from the HREC.

Kind regards,

Ethics Executive Officer

UNIVERSITY *of*
TASMANIA
